# Comparative efficacy and safety of 577-nm diode laser versus 1064-nm Nd: YAG laser for inflammatory acne vulgaris: a split-face randomized study

**DOI:** 10.1007/s10103-025-04654-x

**Published:** 2025-10-10

**Authors:** Essamelden M. Mohamed, Hazem L. Abdel-Aleem, Ghadeer M. H. Elazab, Mahmoud A. Rageh

**Affiliations:** 1 Department of Dermatology, Faculty of Medicine, Al-Azhar University, Assiut, Egypt; 2 Department of Dermatology, Faculty of Medicine, Al-Azhar University, Cairo, Egypt

**Keywords:** Acne vulgaris, Laser therapy, 577-nm diode, Nd:YAG laser, Yellow laser

## Abstract

Acne vulgaris is a common inflammatory skin disorder with physical and psychosocial impact. Laser therapies offer targeted, non-pharmacologic treatment options. We aimed to compare the efficacy and safety of the 577-nm diode laser versus the 1064-nm Nd: YAG laser in treating inflammatory acne vulgaris. In this prospective, split-face randomized trial, 52 patients underwent three sessions of laser treatment at two-week intervals. One side of the face was treated with a 577-nm diode laser and the other with a 1064-nm Nd: YAG laser. Outcomes included inflammatory lesion counts, Acne Severity Index (ASI), patient satisfaction, and adverse effects. Both lasers significantly reduced inflammatory lesions (*p* < 0.001), with no significant difference between groups (*p* > 0.05). Excellent ASI response was observed in 26.9% (diode) and 28.8% (Nd: YAG) treated sides. Satisfaction scores were comparable (2.5 ± 0.6 vs. 2.6 ± 0.5; *p* = 0.59). Adverse events were mild and transient, with no serious complications. The 577-nm diode and 1064-nm Nd: YAG lasers are equally effective and well-tolerated for inflammatory acne, offering safe alternatives to conventional therapies.

## Introduction

Acne vulgaris (AV) is a chronic inflammatory disorder of the pilosebaceous unit affecting up to 90% of adolescents and frequently persisting into adulthood [1]. Its multifactorial pathogenesis includes increased sebum production, follicular hyperkeratinization, *Cutibacterium acnes* colonization, and inflammation—resulting in comedones, papules, pustules, nodules, and cysts [2].

Though not life-threatening, AV significantly impacts quality of life, often causing anxiety, depression, and reduced self-esteem [3]. Conventional treatments—topical agents, antibiotics, hormonal therapies, and isotretinoin—are effective but limited by delayed onset, adverse effects, resistance, and patient noncompliance. Other treatment options include hormonal agents like spironolactone, chemical peels, light-based therapies, and combination regimens tailored to acne severity and patient profile [4–6].

Recently, laser and light-based therapies have gained popularity as adjuncts or alternatives, targeting key pathogenic factors with faster results and fewer systemic effects [7]. The 1064-nm long-pulsed Nd: YAG laser penetrates deeply, thermally targeting sebaceous glands and vasculature, with established efficacy and safety, especially in darker skin types [8].

In contrast, the 577-nm yellow diode laser selectively targets oxyhemoglobin, offering precise vascular and anti-inflammatory effects with reduced downtime [9]. Despite promising early data, its role in acne remains underexplored.

This study aimed to compare the clinical efficacy, safety, and tolerability of the 577-nm diode laser versus the 1064-nm Nd: YAG laser in a split-face randomized trial for inflammatory acne vulgaris. The design minimizes inter-patient variability and allows direct intra-individual comparison of both modalities.

## Patients and methods

### Study design and ethical approval

This prospective, split-face, randomized clinical trial aimed to compare the efficacy and safety of a 577-nm diode laser versus a 1064-nm long-pulsed Nd: YAG laser in treating inflammatory acne vulgaris. The protocol adhered to the Declaration of Helsinki and was approved by the local Institutional Review Board. Informed written consent was obtained from all participants.

### Study population

Fifty-two patients with predominantly inflammatory acne lesions (papules, pustules, and nodules), which were symmetrically distributed on both sides of the face, were enrolled.

Patients were excluded if they had received isotretinoin within the past six months or had used topical or systemic acne treatments—including antibiotics, retinoids, or corticosteroids—within the preceding month, in order to prevent residual treatment effects that could influence outcomes. Additional exclusion criteria included a history of photosensitivity or photosensitive dermatoses, keloid or hypertrophic scar formation, pregnancy or lactation, or the presence of active skin infections or inflammatory dermatoses on the face. Patients with a history of laser therapy for acne or recent cosmetic procedures on the face were also excluded.

Detailed dermatologic and medical histories were recorded, including age of onset, acne duration, prior treatments, menstrual history (females), PCOS, hirsutism, and family history of acne.

### Laser devices and treatment protocol

Each patient underwent three treatment sessions at two-week intervals. One side of the face was randomly assigned (via coin toss) to receive a 577-nm high-output pulsed diode laser (Asclepion, Jena, Germany), while the opposite side was treated with a 1064-nm long-pulsed Nd: YAG laser (Synchro HP, Deka, Italy). Laser settings were standardized: the 577-nm diode laser was delivered with a pulse duration of 30 ms, fluence of 18 J/cm², scanner mode, and a single pass. The 1064-nm Nd: YAG laser was administered using a 40 ms pulse duration, 32 J/cm² fluence, 7 mm spot size, in non-contact single-pass mode.

Topical anesthesia using a eutectic mixture of lidocaine 2.5% and prilocaine 2.5% cream (EMLA^®^, AstraZeneca) was applied to the entire face for 45 min prior to each session. Pre-treatment preparation included gentle facial cleansing with an alcohol-free cleanser. Protective eyewear was worn by both patients and clinical staff, and an external cold air-cooling system (Cryo 6, Zimmer MedizinSysteme, Germany) was used during laser delivery to improve patient comfort and reduce epidermal damage. Following laser treatment, a cold compress with ice was applied for 20 min. All participants were instructed to apply broad-spectrum sunscreen daily, and to avoid any concurrent topical or systemic acne treatments during the study period.

### Outcome measures and follow-up

Primary outcomes included changes in inflammatory lesion counts (papules, pustules, and nodules) on both facial sides, assessed at baseline and monthly for 3 months following the final treatment session. Lesion counts were performed manually by two independent dermatologists who were blinded to the treatment allocation. Any discrepancies were resolved by consensus. The Acne Severity Index (ASI) was calculated, and therapeutic response was classified as excellent (> 75% improvement), good (50–75%), moderate (25–49%), or poor (< 25%) [8]. Patient satisfaction was measured using a four-point scale (0 = not satisfied to 3 = very satisfied). Adverse events, including erythema, pigmentary changes, and scarring, were recorded throughout the study.

### Adverse events and safety monitoring

All patients were monitored closely for immediate and delayed adverse events during and after each treatment session, as well as at follow-up visits. Participants were instructed to report any discomfort, erythema, blistering, pigmentary changes, scarring, or signs of infection. Pain severity during treatment was assessed using a 10-point visual analog scale (VAS), where 0 indicated no pain and 10 indicated the worst imaginable pain. Pain scores were further categorized as mild (1–3), moderate (4–6), or severe (7–10).

### Statistical analysis

Data were analyzed using IBM SPSS Statistics (v23.0). Continuous variables were expressed as means ± SD and analyzed using paired t-tests or repeated measures ANOVA. Categorical data were presented as frequencies/percentages and compared using Chi-square or Fisher’s exact tests. A p-value < 0.05 was considered significant. Sample size was calculated with Stata/IC 16.1, assuming 80% power and α = 0.05 to detect a clinically meaningful difference in lesion count reduction.

## Results

A total of 52 patients (47 females and 5 males) with inflammatory acne vulgaris completed the study. The mean age was 21.98 ± 2.21 years (range: 16–25 years). Most participants had Fitzpatrick skin phototype IV (*n* = 33; 63.5%), and 40 patients (76.9%) reported a family history of acne. Among female participants, 38 (73.1%) had regular menstrual cycles, while polycystic ovary syndrome (PCOS) and hirsutism were reported in 6 (11.5%) and 5 (9.6%) cases, respectively. Based on baseline evaluation, acne severity was classified as mild in 38 patients (73.1%), moderate in 10 (19.2%), and severe in 4 (7.7%) (Table 1).

**Table 1:** Clinical and demographic data of studied patients

Age (years):	
(Range) Mean ± SD	(16–25) 21.98 ± 2.21
Gender: n (%)	
Male	5 (9.6)
Female	47 (90.4)
Skin photo type: n (%)	
III	19 (36.5)
IV	33 (63.5)
Menstrual history: n (%)	
Regular	38 (73.1)
Irregular	10 (19.2)
No menses	4 (7.7)
Duration (years):	
(Range) Mean ± SD	(1–7) 3.63 ± 1.34
Family history: n (%)	
Positive	40 (76.9)
Negative	12 (23.1)
Associated disorders: n (%)	
PCO	6 (11.5)
Hirsutism	5 (9.6)
No association	41 (78.8)
Acne severity: n (%)	
Mild	38 (73.1)
Moderate	10 (19.2)
Severe	4 (7.7)

At baseline, there was no statistically significant difference in lesion counts between the right and left sides of the face (*p* > 0.05). Both the 577-nm diode laser and the 1064-nm Nd: YAG laser led to a significant reduction in inflammatory lesions—including papules, pustules, and nodules—compared to baseline (*p* < 0.001), with sustained improvement maintained through the 3-month follow-up. No new lesions developed on either side of the face during the study (Table 2).

**Table 2:** Comparison of baseline and post-treatment acne lesions numbers

		Baseline	After 1 month	After 2 months	After 3 months	p-value
		(Range) Mean ± SD				
Acne Papules	Nd:YAG	(1–20) 6.6 ± 4.2	(1–17) 4.9 ± 5.1	(0–15) 3.7 ± 3.8	(0–7) 2.4 ± 1.9	p1 = 0.001* p2 = 0.6
	Diode	(0–27) 6.5 ± 4.8	(0–20) 4.6 ± 5.5	(0–10) 2.3 ± 2.4	(0–4) 0.9 ± 1.5	
Acne Pustules	Nd:YAG	(0–8) 1.8 ± 1.8	(0–8) 1.4 ± 2.03	(0–3) 0.6 ± 0.8	(0–4) 0.3 ± 1.02	p1 = 0.001* p2 = 0.8
	Diode	(0–10) 1.8 ± 1.9	(0–7) 1.0 ± 1.8	(0–4) 0.6 ± 0.9	(0–2) 0.3 ± 0.5	
Acne Nodules	Nd:YAG	(0–9) 1.8 ± 1.9	(0–8) 0.4 ± 1.3	(0–6) 0.3 ± 0.9	(0–5) 0.4 ± 0.9	p1 = 0.001* p2 = 0.68
	Diode	(0–8) 1.1 ± 1.9	(0–6) 0.2 ± 0.9	(0–4) 0.3 ± 0.7	(0–2) 0.1 ± 0.4	

ANOVA test was used, * p-value < 0.05 is significant, p1 = Change in number of acne lesions between baseline and after treatment, p2 = Change in number of acne lesions between Nd-YAG and Diode lasers

**Fig. 1:**
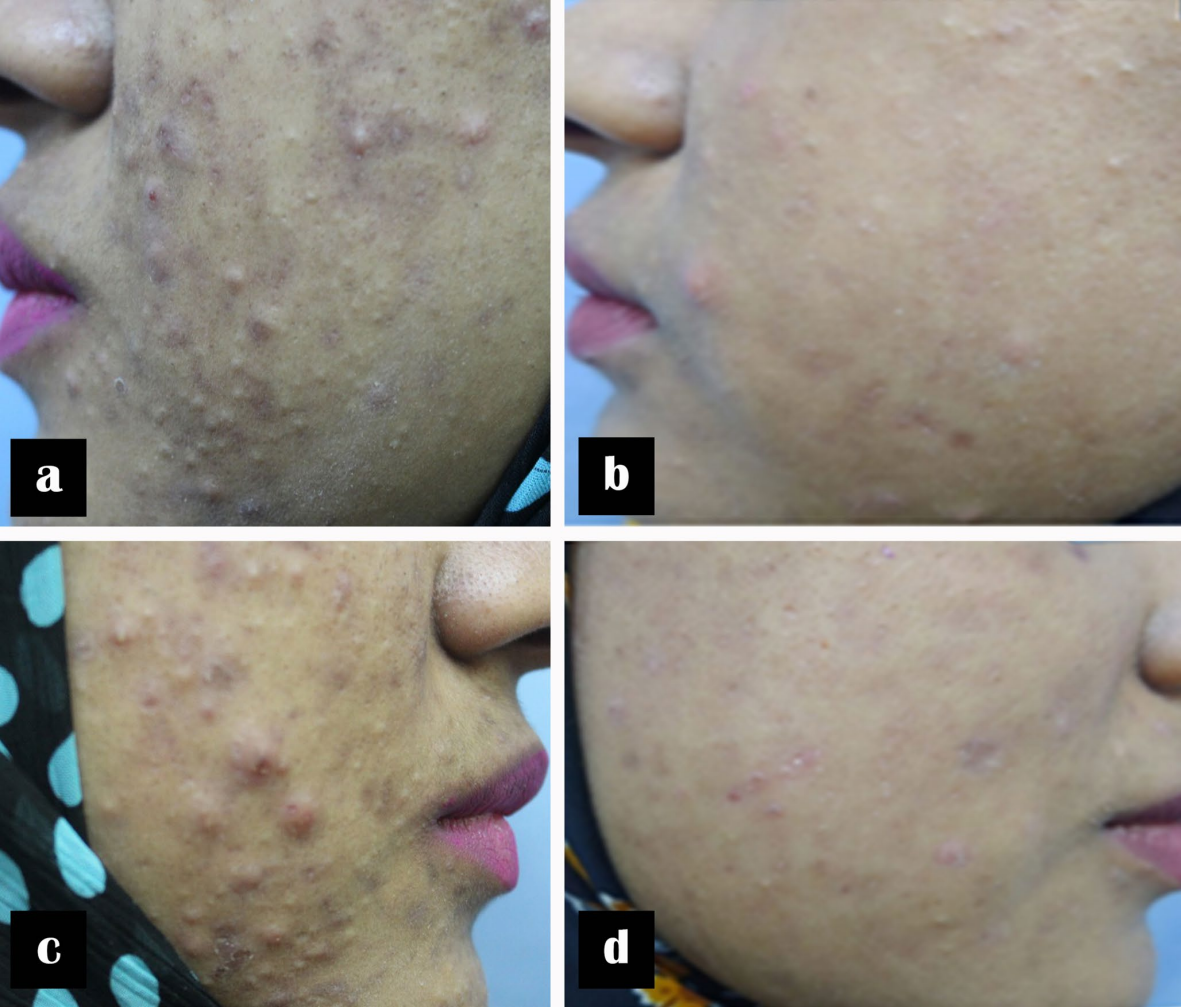
A 21-year-old female with severe inflammatory acne of 6 years’ duration. Clinical photographs before and after three laser treatment sessions. (**a**, **b**) Left side before and after treatment with 1064-nm Nd: YAG laser; (**c**, **d**) right side before and after treatment with 577-nm diode laser

**Fig. 2:**
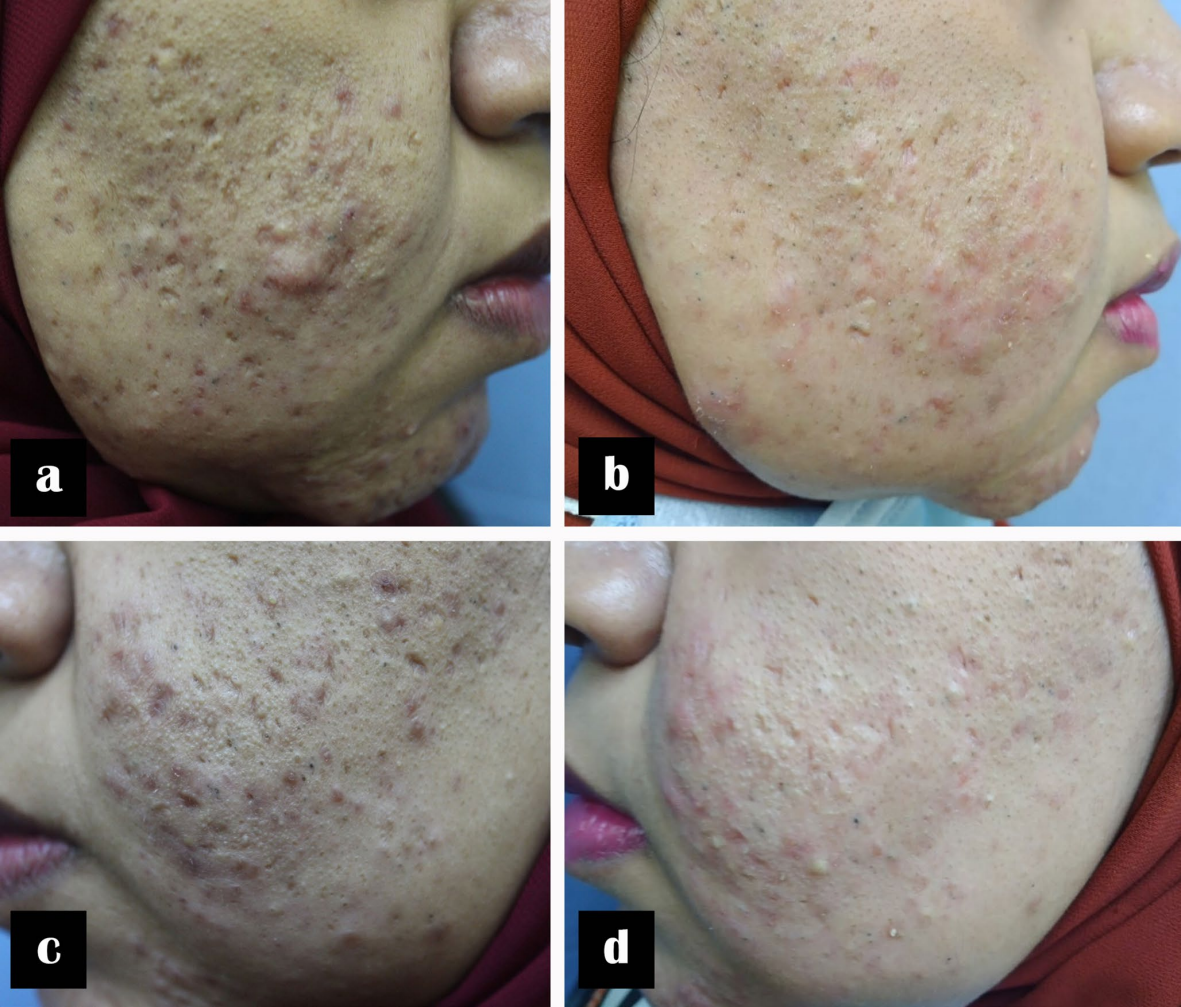
An 18-year-old female with moderate inflammatory acne of 2 years’ duration. Clinical photographs before and after three laser treatment sessions. (**a**, **b**) Right side before and after treatment with 1064-nm Nd: YAG laser; (**c**, **d**) left side before and after treatment with 577-nm diode laser

Lesion clearance was noticeable after the first session and progressively improved with each treatment. By the end of the study, more than 90% of patients showed clinical improvement on both facial sides. According to the Acne Severity Index (ASI) response categories, an excellent response was observed in 26.9% of diode-treated sides and 28.8% of Nd: YAG-treated sides. A good response occurred in 38.4% and 40.4% of the respective groups, while a moderate response was noted in 25.0% and 23.1%. A poor response was observed in 7.7% of patients in both treatment groups. There was no statistically significant difference in ASI score changes between the two laser modalities (*p* > 0.05), indicating comparable therapeutic efficacy (Table 3; Figs. 1 and 2)

**Table 3:** Comparison of the degrees of improvement (according to the acne severity Index), pain, and adverse events between Nd-YAG and Diode laser

	Nd:YAG laser	Diode laser	*p*-value
	*n* (%)		
Degree of improvement			
Excellent improvement	15 (28.8)	14 (26.9)	0.82†
Good improvement	21 (40.4)	20 (38.4)	
Moderate improvement	12 (23.1)	13 (25)	
Poor improvement	4 (7.7)	4 (7.7)	
Pain during sessions			
No pain	40 (76.9)	39 (75.9)	0.80‡
Mild pain	12 (23.1)	13 (25)	
Moderate pain	0 (0.0)	0 (0.0)	
Severe pain	0 (0.0)	0 (0.0)	
Adverse events			
Transient erythema	16 (30.8)	14 (26.9)	0.61†
No adverse events	24 (46.1)	25 (48.1)	

†Chi-square test was used, ‡Fisher’s exact test was used; p-value > 0.05 is non-significant

.

Patient satisfaction was high across both treatment arms. Using a 4-point scale (0–3), the mean satisfaction scores were 2.5 ± 0.6 for the diode laser and 2.6 ± 0.5 for the Nd: YAG laser, with no significant difference between groups (*p* = 0.59).

Transient erythema was observed in 16 patients (30.8%) on the Nd: YAG-treated side and in 14 patients (26.9%) on the diode-treated side. All reported pain was categorized as mild (VAS 1–3) on both sides. No patients experienced moderate or severe pain. Mean VAS pain scores were 0.6 ± 0.8 for the 1064-nm Nd: YAG side and 0.7 ± 0.9 for the 577-nm diode laser side. No adverse events occurred in 24 patients (46.1%) on the Nd: YAG-treated side and in 25 patients (48.1%) on the diode-treated side (Table 3).

All side effects were mild and self-limiting, resolving within a few hours to a maximum of 24 h without intervention. No cases of blistering, prolonged erythema, hyperpigmentation, scarring, or secondary infections were reported. No participants discontinued the study due to adverse effects.

## Discussion

Acne vulgaris remains one of the most prevalent dermatological conditions, especially in adolescents and young adults. Despite the availability of numerous pharmacologic options, concerns regarding side effects, long-term safety, and antibiotic resistance have spurred increasing interest in non-pharmacologic alternatives such as laser and light-based therapies [7].

This study offers comparative clinical evidence on the efficacy and safety of two laser modalities: the 577-nm diode laser and the widely studied 1064-nm long-pulsed Nd: YAG laser. Both significantly reduced inflammatory lesion counts over three sessions, with improvements observed early and sustained throughout follow-up [9–11].

The 577-nm diode laser, part of the yellow light spectrum, is selectively absorbed by oxyhemoglobin and effectively targets vascular inflammation and erythema. Although it is absorbed by melanin, it remains safe for individuals with skin phototypes III–V when used within appropriate parameters. In addition to its vascular effects, it may exert anti-inflammatory and antibacterial actions by generating reactive oxygen species (ROS) that reduce *Cutibacterium acnes* [12]. Mohamed et al. reported significant improvement in papules and erythema using this modality, findings that align with our results and support its clinical utility [9].

In contrast, the 1064-nm Nd: YAG laser penetrates more deeply and primarily targets sebaceous glands, reducing sebum output and bacterial load via photothermal effects [8]. Its low melanin absorption makes it safer for darker skin types, with minimal risk of post-inflammatory hyperpigmentation. Previous studies, including those by Kesty and Goldberg, have shown substantial improvement in inflammatory lesions and comedones with Nd: YAG, findings that are consistent with our observations [11].

Although the two lasers have distinct mechanisms—superficial vascular targeting by the 577-nm laser and deep sebaceous targeting by the 1064-nm Nd: YAG—both achieved comparable clinical results in our study. This may reflect their shared ability to modulate inflammation and reduce microbial load, regardless of penetration depth. In our cohort with mixed lesion morphology, both lasers demonstrated broad efficacy.

Tolerability was high, with only transient erythema and mild pain reported. No patients experienced scarring, hyperpigmentation, or prolonged irritation. Satisfaction scores were similar across both treatment groups, indicating positive patient perception and acceptance.

Other vascular-targeting lasers, including pulsed dye lasers (PDL) and KTP lasers, have demonstrated similar efficacy in reducing inflammatory acne. Seaton et al. observed a 53% reduction in lesions after 595-nm PDL treatment [13], while Baugh et al. reported improvement with 532-nm KTP [14]. Our results extend these findings by supporting the efficacy of the newer 577-nm wavelength.

In addition, emerging technologies, such as the 1726-nm laser, have also demonstrated promising anti-inflammatory and sebosuppressive effects in early clinical trials and may represent future directions in acne therapy [15].

Laser-based modalities represent a valuable option for patients who are unwilling, intolerant of, or unresponsive to conventional pharmacologic therapies. In addition to reducing lesions, they offer cosmetic benefits, including the diminution of post-acne erythema, potentially improving overall patient satisfaction and quality of life.

This study is limited by its relatively small sample size and short follow-up period. Additionally, participants were recruited from a single geographic region, which may limit the generalizability of the findings. Future studies with larger, more diverse populations and objective evaluation tools (e.g., sebumeter, dermoscopy) are warranted.

## Conclusion

Both the 577-nm diode laser and the 1064-nm Nd: YAG laser are effective, safe, and well-tolerated treatments for inflammatory acne vulgaris. There were no statistically significant differences in efficacy, side effects, or patient satisfaction between the two lasers, making either modality a viable non-pharmacological option.

## Data Availability

The data that support the findings of this study are available upon reasonable request.
